# Staff-Care by Chaplains during COVID-19

**DOI:** 10.1177/1542305020988844

**Published:** 2021-03-17

**Authors:** Beba Tata, Daniel Nuzum, Karen Murphy, Leila Karimi, Wendy Cadge

**Affiliations:** Department of Spiritual Care, Mayo Clinic, Rochester, MN, USA; College of Medicine and Health, University College Cork, Cork University Maternity Hospital & Cork University Hospital, Ireland; Weston Hospicecare, Weston-super-Mare, UK; School of Psychology and Public Health, La Trobe University, Melbourne, Australia; Department of Sociology, Brandeis University, Waltham, MA, USA

**Keywords:** Chaplaincy, COVID-19, Spiritual Care, staff support, Pastoral care

## Abstract

The aim of this study was to understand how chaplains delivered spiritual care to staff during the Covid-19 pandemic. The researchers analyzed data collected from an International Survey of Chaplain Activity and Experience during Covid-19 (N = 1657). The findings revealed positive changes that emerged and new practices evolved around the use of technology as useful tools for maintaining contact with staff.

## Introduction

Patients and family members have traditionally been the main focus of spiritual care (Liberman et al., 2020). There is a recognized role for chaplains supporting staff colleagues (UKBHC, 2014). The COVID-19 pandemic has challenged healthcare institutions considerably. The impact of isolation and quarantine are well documented and early studies in Wuhan, China illustrate considerable emotional burden for healthcare staff (Kang et al., 2020; Lai et al., 2020; Leong et al., 2004). The positive impact of chaplaincy support for healthcare staff in high-stress contexts has been recognized in various studies and there is evidence that chaplains have found innovative ways to provide staff support (Byrne & Nuzum, 2020; Greenberg et al., 2020).

### Tested Methods of Staff Care

Various staff care initiatives have been employed and studied in recent years. The post-code pause addresses the lack of a formal debriefing process following a trauma event, addressing spiritual and psychological needs (Copeland & Liska, 2016). A Scottish healthcare study emphasized the increasingly positive role of chaplains in supporting staff on a regular basis, as well as developing an integrated educational role (Butler & Duffy, 2019). Opportunities to gather staff together for remembering patients and families are also useful and supportive (Murphy & Whorton, 2017). An ‘on-the-job mindfulness-based intervention’ for pediatric ICU nurses was tested in 2012. This study showed positive results of 5-minutes of mindfulness instruction before shifts with all of negative-correlated emotions decreasing, and positive ones increasing (Gauthier et al., 2015).

Three studies have illustrated that interpersonal relationships between staff have been shown to provide secondhand staff care, and that when chaplains are integrated members of the care team and staff know how to appropriately use their services, the benefits can be tangible (Hemming et al., 2016; Liberman et al., 2020; Taylor et al., 2015). Finally, chaplain-led spiritual care groups, as seen in the “Programmatic Self Care in an Outpatient Setting” study is evidence of effective ways in which chaplains provide spiritual care to hospital staff (King et al., 2005). In conjunction with this study, an ‘After Book’ was created to provide staff with information about patients on discharge and when death had occurred, providing helpful information to enable reflection (King et al., 2005). The literature also identifies barriers for chaplains when tasked with providing staff care in a hospital setting. They are infrequently given protocol for this care, and hospital staff are rarely taught how to utilize chaplaincy services, leading to a lack of standardization (Hemming et al., 2016).

The aim of this study was to understand how chaplains delivered spiritual care during the Covid-19 global pandemic. This article focuses on staff care asking whether, and how chaplains provided staff care in the midst of the pandemic, what factors led some but not other chaplains to do staff care, what did the care consist of, what effect did chaplains think it had.

## Methods

An International Survey of Chaplain Activity and Experience during Covid-19 was disseminated electronically through professional chaplaincy networks in Europe, North American, Australia and more minimally in Asia, Africa and South America. Inclusion criteria were that participating chaplains were currently working in healthcare facilities. Chaplains who volunteered (i.e. were not hired directly by the healthcare facility) were excluded. All participants provided informed consent. The study was approved by KU Leuven research ethics committee.

### Data Collection and Analysis

The 40-question survey was predominantly closed-ended questions. A few open-ended questions were asked that enabled respondents to write in what was new, what was effective, what was lost, and what had changed in chaplain practices during the pandemic.

Data about staff care were analyzed by the research team of three chaplaincy researchers; a sociologist; and a quantitative research analyst. Descriptive analysis and inferential analysis were conducted using SPSS (v 26). To determine patterns in which chaplains conducted staff care, bivariate analysis was used with a set of individual and organizational predictors described in current literature on the subject. Logistic regression was used to predict the effects of some demographic and organizational factors on the likelihood of chaplains providing staff support adapting a 0.05 criterion of statistical significance. Qualitative data from the open-ended questions was analyzed by four members of the research team using thematic analysis. Key themes were discussed between the research team. Given the volume of data, the team then divided the questions, each of the four researchers coded one question, and the team continually discussed the findings and analysis.

## Findings

One thousand six hundred and fifty-seven people participated (n = 1657); 666 from Europe, 730 from North America and Canada, 202 from Australia, 12 from Asia, and 8 from Africa and South America. The survey was completed online between May 18, 2020 and May 29, 2020. Respondents worked as professional chaplains in healthcare facilities and were of all ages, genders, and faiths.

Table 1 outlines the demographic and organizational characteristics of participating chaplains. The majority of participants were female (55.5%), worked in a hospital setting (60.5%), worked as part of a spiritual care team (74.4%), and belonged to a professional association for chaplains (71.6%). The mean age was 58 years and the mean years of service as a chaplain was 14. Religiously, respondents identified as Protestant (55.8%), Catholic (24.9%), Muslim (0.7%), Buddhist (1.1%), Hindu (0.4%), Jewish (3.9%), Humanist (2.8%), Other (4.6%), (5.9%) did not respond to this question.

Table 2 describes the extent to which chaplains conducted staff care and the frequency with which they did so. As demonstrated, most of the time chaplains were asked to provide staff care (84%). Organizations involved the chaplains in staff care most of the time (82%) while only over half of the time (60%) the staff care was provided by the chaplains.

**Table 1.** Demographic and organizational characteristics of the chaplains.^a^

	Frequency	Valid percent
Gender:		
Male	660	41.0
Female	920	57.2
Other/Prefer not to say	29	1.8
Qualifications:		
Under graduate	257	16.8
Post graduate	1275	83.2
Work setting during the pandemic:		
Nursing home	199	19.4
Hospital	826	80.6
Member of a professional association:		
No	323	21.4
Yes	1187	78.6
Religion:		
Christian Protestant	924	59.4
Christian catholic	412	26.5
Others	220	14.1
Continent:		
Australia	202	12.6
Europe	666	41.7
North America	730	45.7
Access to COVID patients:		
Yes	645	51.8
No	601	48.2
Access to non-COVID patients:		
Yes	1049	85.8
No	173	14.2
Age (years)	Mean (53.9)	SD (11.6)

^a^*n* varies from 1025–1598 due to some missing data.

To determine patterns in which chaplains conducted staff care, we conducted bivariate analysis. The dependent variable was “During the pandemic, did you spend time supporting staff?” Independent variables included membership of a professional association, religion, continent, access to COVID and non-COVID patients, work setting, gender, etc.

Table 3 describes the logistic regression conducted and to further explore predictors of providing staff support. Protestant chaplains were more likely (93%) to provide staff support than chaplains from other religious backgrounds (P < 0.05). Although significant, chaplains in Europe indicated less staff support than their peers in Australia, chaplains in North America were two times more likely to provide staff support during the pandemic. Chaplains with access to non-COVID patients were 2.41 times more likely to provide staff support (141%) than those with no access. Finally, those working at a hospital setting were more likely (66%) to provide staff support than those working at a nursing home setting.

**Table 2.** Support provided for staff by chaplains.^a^

	Frequency	Percent
When asked to provide spiritual care for staff:		
Never/rarely	203	16.1
All/most of the time	1061	83.9
Did your organization involve you in staff care:		
No	214	17.8
Yes	987	82.2
Staff care provided during the pandemic:		
No/rarely/sometimes	544	41.1
Often/all the time	779	58.9

^a^*n* varies from 1222-1264 due to some missing data.

**Table 3.** Binary logistic regression analysis of predicting staff support by chaplains by demographics, individual, organizational factors.

Independent variables	*B*	SE	*Z* ratio (Wald)	Sig.	Odds ratio	95% CI for odds ratio	
						Lower	Upper
Religion:			14.37	0.001			
Others							
Christian protestant	0.65	0.26	6.0	**0.01**	1.93	1.14	3.25
Christian Catholic	0.033	0.28	0.01	0.91	1.03	0.58	1.81
Continent:			53.10	0.001			
Australia							
Europe	−0.630	0.269	5.85	**0.01**	0.52	0.31	0.88
North America	0.699	0.286	7.08	**0.008**	2.01	1.21	3.67
Access to COVID (YES)	0.365	0.195	3.94	0.04	1.46	1.00	2.12
Access to non-COVID (YES)	0.867	0.352	6.61	**0.01**	2.41	1.23	4.73
Member of a professional association (YES)	0.241	0.212	1.90	0.16	1.33	0.88	2.00
Work Setting During Pandemic (Hospital vs nursing)	0.51	0.20	5.99	**0.01**	1.66	1.10	2.50
Constant	−1.50	0.45	11.06	0.001	0.22		
Model	*χ*^2^ = 11.54, *p* > 0.05						
Pseudo	*R*^2^ = 0.21						

Note: The dependent variable in this analysis is staff support provided by chaplains.

coded so that 0 = none/rarely/sometimes and 1 = often/all the time. The Omnibus Tests of was significant (*p* < 0.05); Hosmer and Lemeshow was not significant showing a good fit for the tested model (*P* > 0.05).

Many chaplains noted that their role expanded during the pandemic and they had increased visibility amongst staff colleagues. Respondents noted a greater sense of appreciation and knowledge of what chaplains do and bring as fellow professionals to the multidisciplinary healthcare team. Chaplains experienced ‘fluidity in role’ where they entered new areas for exercising ministry such as presence at team meetings, inclusion in decision making in staff support/planning. These responses gave the impression that the chaplain’s role with staff support and spiritual care was taken to a new level. Wearing ‘scrubs,’ getting involved in practical care, *‘getting my hands dirty,’* as well as showing willingness to be available and vulnerable, all contributed to effective teamwork and a high standard of chaplaincy presence where possible. Chaplains spoke of a new realization and appreciation by staff of the value of spiritual support for themselves and how the chaplain was an available resource for their own well-being.

In many institutions where chaplains were asked to work remotely, they reported that their inability to provide in-person ministry and face-to-face contact with staff during the pandemic was a big loss. Many of the chaplains grieved the “unplanned interactions, moments of prayer, or encounters” that allowed them to listen to staff members’ “daily joys and complaints” and promote wellbeing. Chaplains from across the continents grieved the loss of human contact especially actions such as “a simple touch on the shoulder or holding a hand as a sign of comfort when praying, sharing a hug when staff is distraught, as well as providing a “supportive arm or shoulder for staff to cry on.” Chaplains lamented that this lack of human contact led to the loss of “the spiritually healing significance of touch,” the “hands on” approach of spiritual care; the physical aspect of “journeying with, and the feeling of being alongside the medical staff.” The chaplains who were only able to work remotely reported the “loss of a sense of solidarity” with staff and felt like they had lost some of the strong “interdisciplinary relationships” that they had formed prior to the pandemic. As a result, there was an “interruption in continuity of care and a loss of the sense of team cohesion.”

While many responses indicated that face-to-face contact was more comfortable and familiar, chaplains embraced new means of making connections which worked effectively. Doing work from a distance through phone calls and zoom was new as was regularly wearing PPE for those serving staff in person. Some chaplains indicated a need among staff to speak about deeper personal concerns as time progressed. Fear for the future, for family and friends’ health as well as facing their own mortality and becoming ill. Many said staff anxiety was heightened and there was, “greater need to support staff who were stressed, anxious, grieving many losses.” Some received more referrals for staff while others created new ways to access and talk with staff who were, “significantly more willing to talk.” At one hospital chaplains created a team to “call most of the staff on the phone” while at another they made a point to be “present in staff support hubs and quiet rooms.”

For most chaplains, some kind of ‘telechaplaincy’ was quickly established to provide ‘safe and effective’ means of supporting staff in a new way. While many responses indicated that face to face was more comfortable and familiar, chaplains embraced new means of making connections which worked effectively. There was also an indication that online connections would continue to link teams who rarely met face to face. Time saving and more open participation were also factors that would influence using effective technology in the future.

In addition to technology, new approaches to staff included wellness surveys, debriefs with staff, educational resources on self-care, anxiety and stress, and well as “wellbeing hubs with food and drink and treats, lots of resources for them to use with patients, knitted or crocheted hearts for staff or patients.” Chaplains placed “positive messages around the hospital” and in many settings created regular videos, social media posts and/or thank you notes to share. At one hospital they made, “blessing jars for all staff areas in the hospital containing words of encouragement and reflection.”

In some settings, chaplains did this work on their own while in others they were part of teams supporting staff. At one hospital the chaplains worked, “closely with colleagues from other disciplines to provide a coordinated approach to staff care and support.” Several commented that they felt more a part of staff teams through this work and had a “greater sense of 'team' within the staff groups I serve and a feeling of being a part of those teams.” A more inclusive understanding of spiritual care in the care team developed among staff who often ‘see us differently’ than other allied health staff.

A minority of chaplains experienced negative impacts of COVID-19 on their ability to provide staff care. This ranged from an awareness of the lack of chaplaincy presence amongst staff colleagues during COVID-19. For some chaplains they felt that their ministry was undervalued by their healthcare system. Where chaplains were prevented from being physically present this was experienced as a negative impact on their capacity to provide care for staff and also a recognition that this was felt by staff too who were also isolated in the supports available to them. It also prompted reflection on the value or priority of spiritual care in pandemic. For some chaplains they expressed that they were not included and, in a few cases, excluded from staff support structures. Some chaplains felt that it was too early in the pandemic to evaluate changes in care as a result of COVID-19.

## Discussion

This study presents a global picture of chaplaincy contribution to staff support during a time of trauma and distress. It is a unique attempt to explore how, chaplains have responded within their organizations. The data has identified how chaplaincy is valued, or disregarded in some instances, as a resource for staff support. Through the pandemic, creative spiritual care became available to staff which created new understanding of the chaplain’s role and spiritual support as a whole. The impact of these new relationships and the depth of sharing that has occurred should define a turning point for chaplaincy to be more integrated into the healthcare team, and therefore, be available for staff support. A repeated theme of loss was lack of face to face contact with staff, a shared lack of being able to offer human touch to patients and spontaneity of contact which builds relationships between staff and chaplains. Positive changes emerged as chaplains were more visible and had time to spend with staff, sharing concerns and listening to one another. New practices evolved around the use of technology as useful tools for maintaining contact with staff.

## Strengths and Limitations

This study is the first global study to capture the provision of staff care by chaplains in a pandemic. The data were collected through a robust research method which gave researchers strong qualitative and quantitative results including the lived experiences of participants. Identifiable weaknesses of the study are that the data were collected relatively early in the pandemic, so is limited to a particular moment in time. As this was an online study disseminated though professional chaplaincy networks it is a weakness of the study that it was not possible to calculate the response rate. Nonetheless, the high numbers of participating chaplains alongside the geographical and religious breadth of participating chaplains is a strength in this global study.

## Conclusions

This study offers some thoughtful material for recommendations in the ongoing pandemic situation.

- Chaplains are, on the whole, becoming more integrated into care teams where previously there had been division or poor understanding of the chaplain’s role. Chaplains can maintain a visible presence among staff and build on the experience so far.
- The contribution of chaplains to care teams has been highlighted by this study and the results can be shared with organizations, thus recognising the place of the chaplain in offering staff care.
- Chaplains documented the benefit to patients as a result of staff having a better understanding of spiritual care. Having time to talk to staff and becoming more visible, enabled staff to be more confident in referring patients to chaplains.
- Chaplains embraced technology as a means of offering staff support and felt that this would be a useful tool to use in the future.

There are, however, questions for the future that emerge as a result of the study.

- Social distancing will be normal safe behaviour for the immediate future. As indicated in this study, the impact of physical distance being maintained has been felt to be tremendously negative in terms of offering support. The long-term effects of working with physical distance will be a subject for discussion.
- The study indicates there is a strong correlation between effective communication with staff and chaplaincy support of patients. As face to face handover sessions or staff encounters become less frequent, will chaplains be able to maintain visibility and promote the place of spiritual care with the wider team.

The pandemic continues to be a huge challenge for healthcare staff; but this study shows a willingness by chaplains to offer valuable and beneficial staff support, as well as demonstrating their place in the team in ways previously not noticed or valued.
